# Plasma Concentration of Irisin and Brain-Derived-Neurotrophic Factor and Their Association With the Level of Erythrocyte Adenine Nucleotides in Response to Long-Term Endurance Training at Rest and After a Single Bout of Exercise

**DOI:** 10.3389/fphys.2020.00923

**Published:** 2020-07-31

**Authors:** Ewa Rodziewicz, Magdalena Król-Zielińska, Jacek Zieliński, Krzysztof Kusy, Ewa Ziemann

**Affiliations:** ^1^Department of Physical Therapy and Biological Regeneration, Gdańsk University of Physical Education and Sport, Gdańsk, Poland; ^2^Department of Physical Education and Lifelong Sports, Poznań University of Physical Education, Poznań, Poland; ^3^Department of Athletics, Strength and Conditioning, Poznań University of Physical Education, Poznań, Poland

**Keywords:** adenylate metabolites, purine degradations, long-term endurance training, lactate, exerkines

## Abstract

The study aimed to assess the effect of a single bout of incremental exercise on irisin and BDNF plasma concentrations as related to erythrocyte purine nucleotides concentration at rest and after exercise. Master endurance master athletes (training experience 38 ± 6 years) and a group of untrained participants completed a single bout of progressive incremental exercise test until exhaustion. The dual-energy x-ray absorptiometry and blood collection were performed. Blood was taken twice at rest and 10 min after exercise. Concentrations of ATP, ADP, and AMP were assessed in the erythrocytes. Hypoxanthine and uric acid were determined in plasma using the high-performance liquid chromatography. Plasma concentrations of irisin and BDNF were assessed through the immunoenzymatic method. The ATP level, ATP/ADP ratio and AEC value were significantly higher in the athletic group. A significantly higher concentration of BDNF was it also noted in the trained group that correlated with the erythrocyte energy status at rest. The single session of exercise induced a significant increase in ATP erythrocyte levels in both groups. Both exerkines significantly correlated at rest with red blood cell adenine nucleotides and degradation products (BDNF positively and irisin negatively). The blood concentration of BDNF and irisin, in response to exercise, was not significantly different between groups. Obtained data revealed a higher erythrocyte energy status and lower purine degradation products concentration in master athletes. Also resting plasma exerkines differed substantially between groups. In conclusion, long-term training resulted in exercise adaptation reflected by a higher erythrocyte energy status, lower purine degradation products concentration and modified concentration of exerkines (higher BDNF and lower irisin blood concentrations). Therefore, we consider the training-induced adaptations in master athletes to be beneficial and significant. The moderate level of physical activity in the untrained group, even if sufficient in terms of general health, did not cause any discernible changes.

## Introduction

Muscle cells are highly metabolically active, mainly to deliver energy at rest and during exercise. Skeletal muscles communicate with other organs by producing and releasing myokines (Pedersen, 2019). One of them is irisin, a myokine newly discovered by Bostrom et al. (2012). It is produced from the fibronectin type III domain containing protein 5 and expressed mainly by skeletal muscles (Bostrom et al., 2012). Physical exercise is one of the main factors stimulating the release of irisin (Bostrom et al., 2012) thus, this protein belongs to muscle-derived exerkines – a group of proteins stimulated by exercise (Safdar and Tarnopolsky, 2018).

Despite the initial controversy around irisin, many years of research have since well documented its function. The major action of irisin is the conversion of white adipose tissue into brown adipose tissue by increasing the expression of mitochondrial uncoupling protein 1, thereby increasing energy consumption, thermogenesis, and promoting weight loss (Novelle et al., 2013). It has been also proposed that irisin plays a key role in regulating glucose and lipid metabolism in adipose tissue and skeletal muscles (Lee et al., 2015; Xin et al., 2016). Irisin is further suggested to improve glucose homeostasis by reducing insulin resistance (Perakakis et al., 2017). In addition, it can be a regulator of the cross-talk between muscles and bones. It prevents bone loss by enhancing osteoblast differentiation through the Wingless (Wtn)/β-catenin pathway, increasing the osteoblasts numbers and suppressing osteoclast formation (Anastasilakis et al., 2019). The physiological concentration of circulating irisin is affected by several factors such as age (Huh et al., 2014), metabolic syndrome, insulin resistance (Park et al., 2013), and body composition (Huh et al., 2012). Recently published data revealed that irisin can be also considered as a factor modifying adaptive changes in response to training (Fatouros, 2018).

Irisin was found to cross the blood-brain barrier, enter the central nervous system and stimulate the expression of the brain-derived neurotrophic factor (BDNF) (Jin et al., 2018). Thus, BDNF can also belong to the group of proteins stimulated by exercise, called exerkines (Pedersen, 2019). The main functions of BDNF are associated with the regulation of neurogenesis and neuron growth and survival by increasing their resistance to damage (Mattson et al., 2004). BDNF is not only involved in the regulation of neurogenesis but it also affects other tissues and regulates peripheral metabolism. Previous animal studies indicated that BDNF stimulates glucose uptake by the liver and skeletal muscle cells, improves glucose metabolism and increases insulin sensitivity (Marosi and Mattson, 2014).

In recent years, it was suggested that irisin and BDNF are regulators of energy metabolism in different tissues such as brain or muscle tissue. A study conducted by Huh et al. (2012) indicated that circulating irisin is associated with muscle ATP and ADP content and thus regulates muscle metabolism before and after exercise. Moreover, an animal study showed that treatment with irisin improves energy metabolism in the prefrontal cortex by enhancing ATP level and up-regulating ATP level in astrocyte cells via the AMPK pathway (Wang and Pan, 2016). Previous data showed that also BDNF regulates cellular bioenergetics and increases neuronal ATP production (Cheng et al., 2012). Pospieszna et al. (2019) demonstrated that in competitive athletes, an increment in the RBC energy status occurred across an annual training cycle, as assessed by the increments in AEC and ATP/ADP and ADP/AMP ratios. This suggests that red blood cell metabolism adjusts to increased physical requirements of consecutive training phases. Still, there are no data about the association of irisin or BDNF with erythrocyte energy status in human.

Chronic physical training leads to many adaptive changes in blood and RBC, e.g., regulation of oxygen delivery and uptake (Mairbaurl, 2013). Therefore, chronic training as well as a single bout of physical exercise has an impact on erythrocyte energy status and purine nucleotides metabolism in RBC. An *in vitro* study conducted by Berman et al. (1988) indicated that conditions associated with an intense physical exercise (low pO_2_, decreased pH in RBC and increased intracellular inorganic phosphate-Pi concentration) lead to changes in purine nucleotide metabolism: a decrease in ADP together with an increase in erythrocyte ATP and IMP level. However, human studies evaluating the effect of a single bout of exercise and chronic training on purine nucleotides concentration in red blood cells and RBC energy status are limited and inconsistent. On the one hand, changes in ATP concentration with a concomitant decrease in ADP and AMP levels were observed in response to a single session of exercise (Wagner et al., 1991; Dudzinska et al., 2010; Dudzinska et al., 2018). On the other hand, Markiewicz et al. (1980) noted an increase in erythrocyte ATP levels in young swimmers. The erythrocyte ATP levels together with purine metabolites-hypoxanthine (Hx) and uric acid (UA)-are also considered as potential indicators of a training status and physical capacity (O’Brien et al., 2015; Dudzinska et al., 2018). Previous studies indicated that athletes undergoing lifelong sprint training have lower resting and post-exercise plasma Hx and UA concentrations (Zielinski et al., 2019). There is no available data regarding the relationship between exerkines, ATP status and purine metabolites. Diverse forms of exercise can change concentrations of the above biomarkers. In addition, an increase in some exerkines and a drop in its concentration was noted in response to both a single bout of exercise and long-term training (Bostrom et al., 2012; Huh et al., 2014; Norheim et al., 2014; Perakakis et al., 2017; Kim and Kim, 2018). For example, a drop in irisin and BDNF concentration suggests their faster uptake by different tissues. Moreover, changes in blood BDNF concentration in response to exercise do not necessarily reflect changes in BDNF in the brain. They may have also too be associated with an increased uptake by the central nervous system (Knaepen et al., 2010). The recently published paper by Kujach et al. (2019) revealed that changes in peripheral BDNF in response to sprint interval training were accompanied by an increase in lactate levels (LA). Another study pointed out that LA is the “missing exercise factor” inducing BDNF synthesis (El Hayek et al., 2019).

Therefore, this study aimed to assess the effect of a single bout of incremental exercise on irisin and BDNF plasma concentrations and erythrocyte purine nucleotide levels. We also wanted to investigate the link between the two exerkines and erythrocyte purine nucleotides concentration at rest and after exercise.

## Materials and Methods

### Ethics Statement

The study was approved by the local ethics committee at the Poznań University of Medical Sciences and was conformed to the Declaration of Helsinki. Before commencing the study, all participants were informed about the study protocol and related risks and signed written informed consent.

### Participants

Twenty two men aged 58 ± 3.6 years participated in the study. All subjects were healthy, normotensive, with an optimal body mass index and they had no contraindications to exercise. The characteristic of participants is presented in Table 1.

**TABLE 1:** Characteristics of the participants from trained and control group.

**Variables**	**Trained group (*n* = 12)**	**Control group (*n* = 10)**	***p*-value**
	**Mean ± SE**	**Mean ± SE**	
Age (years)	58.64.3	57.42.9	Ns
High (cm)	174.71.5	177.01.3	Ns
Weight (kg)	73.71.9	75.41.6	Ns
BMI (kg⋅m^–2^)	24.20.5	24.00.4	Ns
FM (%)	14.80.9	22.51.1	0.001
LBM (kg)	61.41.9	61.61.5	Ns
VO_2__*max*_ (ml⋅kg^–1^⋅min^–1^)	51.92.7	37.01.8	0.001
HR_*max*_ (bpm)	167.32.4	174.82.4	0.009
Distance (m)	1899.2197.1	1281.5121.8	0.02
Training experience (years)	386.0	0.00.0	0.001
Glucose (nmol⋅L^–1^)	5.20.2	5.10.2	Ns
Insulin (pmol⋅L^–1^)	40.43.0	69.73.0	0.001
HOMA-%B	80.37.8	124.97.5	0.001
HOMA-%S	112.912.2	68.23.5	0.004
HOMA-IR	0.890.1	1.50.1	0.001
Cholesterol (mmol⋅L^–1^)	46.81.6	49.81.1	Ns
LDL (mmol⋅L^–1^)	2.90.2	2.90.1	Ns
HDL (mmol⋅L^–1^)	1.50.1	1.70.1	Ns
TG (mmol⋅L^–1^)	0.750.1	0.850.1	Ns

**Values are given as mean ± SE. Ns, non-significant differences between groups; BMI, body mass index; FM, fat mass; LBM, lean body mass; VO_2__max_, maximal oxygen uptake; HR_max_, maximum heart rate; distance, total distance achieved by participants during the incremental treadmill exercise test; HOMA-%S, insulin sensitivity assessment; HOMA-%B, β-cell function assessment; HOMA-IR, Homeostasis Model Assessment; LDL, low-density lipoprotein; HDL, high-density lipoprotein; TG, triglycerides.**

### Study Design

Participants were assigned to two groups. The highly trained group (*n* = 12, VO_2__*max*_ = 51.9 ± 2.7 mlO_2_⋅kg^–1^⋅min^–1^, training experience = 38.0 ± 6.0 years), consisted of still active endurance master athletes who regularly participated in European and world championships and were classified between 1st and 10th position in their age categories. The second group consisted of untrained participants (*n* = 10, VO_2__*max*_ = 37.0 ± 1.8 mlO_2_⋅kg^–1^⋅min^–1^, without any training experience). All participants performed a single bout of progressive incremental exercise test on a treadmill until exhaustion. Before the test, participants underwent the dual-energy x-ray absorptiometry measurement to assess body composition (Nana et al., 2015). At rest and 10 min after exercise, blood samples were collected. The erythrocyte concentrations of ATP, ADP, and AMP were assessed. Hypoxanthine (Hx) and uric acid (UA) were determined in plasma by the high-performance liquid chromatography method. Plasma concentrations of irisin and BDNF were assessed by the immunoenzymatic method.

### Exercise Protocol

Participants performed a maximal physical capacity test, i.e. an incremental treadmill exercise test until volitional exhaustion to determine maximal oxygen uptake (VO_2__*max*_). Subjects were instructed not to participate in any high-intensity or long-duration training sessions at least 24–48 h before testing. The test was performed in the morning, 3 h after a light breakfast (no coffee or tea). All participants had the same breakfast of calorific value of about 300–400 kcal including water to drink. During all examinations, the ambient temperature was maintained at 20–21°C. The incremental exercise test was performed on a mechanical treadmill (H/PCosmosPulsar, Sports & Medical, Nussdorf-Traunstein, Germany). Initial speed during was set at 4 km⋅h^–1^ and was increased after 3 min to 8 km⋅h^–1^. Then, the treadmill speed increased by 2 km⋅h^–1^ every 3 min until volitional exhaustion. Respiratory parameters were measured (breath by breath) by an ergospirometer (Cortex MetaLyzer 3B, Leipzig, Germany) and analyzed using MetasoftStudio v. 5.1.0 Software (Cortex MetaLyzer 3B; Cortex Biophysik, Leipzig, Germany). The polar Bluetooth Smart H6 (Polar Electro Oy, Kempele, Finland) heart ratemonitor was used to record the heart rate (bpm).

### Sample Collection

Blood samples were collected at two-time points: before and 10 min after incremental exercise. Venous blood was taken from the antecubital vein by a qualified nurse into vacutainer tubes (Vacutainer SST^TM^ II Advance) for serum analysis, the tubes with sodium fluoride to assess glucose concentration and with lithium heparinate (4.9 mL) as an anticoagulant (S-monovette, Sarstedt, Nümbrecht, Germany) to assess erythrocyte purine nucleotides (ATP, ADP, and AMP), plasma Hx and UA.

### Erythrocytes Isolation

To isolate the erythrocyte samples from the lithium heparinate, the tubes were immediately centrifuged (1000 × g, 5 min, 4°C; Universal 320R, Hettich Lab Technology, Tuttlingen, Germany). Buffy coat and plasma were removed and then plasma was frozen at −80°C until the analysis. Next, erythrocytes were washed three times with buffered 0.9% NaCl solution and centrifuged (1000 × g, 5 min, 4°C). After the last wash, the erythrocyte pellet was resuspended with a small volume of PBS. The erythrocytes were then collected in Modulohm glass capillaries (volume 20 μL, length 75 mm) to obtain hematocrit (Htc) values. Then, isolated erythrocytes were deproteinized with a volume of 1.3 mol/L HClO4, mixed, and centrifuged at 16,000 × g for 5 min at 4°C. The neutralization of the supernatant (600 μL) was performed with 130–160 μL of 3 mol/L K_3_PO_4_ (to pH 5–7). The samples were centrifuged again in the same way as above. The supernatant was stored at −80°C until the analysis.

### Erythrocyte Purine Nucleotides Concentration

The purine nucleotide (ATP, ADP, AMP, and IMP) concentrations were assessed in erythrocytes using high-performance liquid chromatography with UV-VIS detection (Merck-Hitachi/Agilent, Tokyo, Japan/Santa Clara, CA, United States) according to the method used and described in details by Smolenski et al. (1990) and Pospieszna et al. (2019). To determine ATP, ADP, and AMP levels, the aliquots of 100 μL were injected into the sample loop. Purine nucleotides were separated using a gradient elution system at a 1 mL/min flow rate. Peaks were detected by absorbance at 254 nm. The concentrations of nucleotides being determined were expressed in relation to erythrocyte volume and expressed as μmol/L RBC.

The values of adenylate energy charge (AEC) were calculated using the following formula (Dudzinska et al., 2006):

$$\text{AEC}=\frac{\left[\text{ATP}\right]+0.5\,\,\left[\text{ADP}\right]}{\left[\text{ATP}\right]+\left[\text{ADP}\right]+\left[\text{AMP}\right]}$$

### Plasma Purine Metabolites (Hx, UA) Concentration

The concentration of purine metabolites was assessed by high-performance liquid chromatography method in neutralized plasma using UV-VIS detection (Merck-Hitachi/Agilent, Japan/United States) according to the method previously described in details by Smolenski et al. (1990) and used by our team (Zielinski et al., 2019). The system contains high-pressure gradient pump L-6200, 1050 diode array detector and autosampler AS 2000A with a thermostatic cooler set at 4°C. Separations were achieved by the analytical column BDS Hypersil C18, 150 mm × 4.6 mm × 3 μm (Thermo Finnigan, United States) protected with guard column 20 mm × 4 mm (Phenomenex, United States). Quantitative analysis was conducted using the ChemStation data system (Agilent, United States) operating on a PC. The substances identification was based on comparison retention times with standards of Hx and UA. The measurement was done at wavelength 254 nm for Hx and 280 nm for UA by comparison with external standards. The within-run/between-run %CVs were 3.1/4.1, 3.3/4.4 and 2.7/3.2% for Hx, X and UA, respectively.

### Plasma BDNF and Irisin Concentration

All of the samples were immediately placed at 4°C and, within 10 min, the samples were centrifuged at 2000 × g for 10 min at 4°C. The separated plasma samples were frozen and stored at −80°C until later analysis. Plasma BDNF concentration was determined before and after exercise by immunoassay method using Elisa Kit (R&D, United States&Canada, catalog no. DBD00) according to the manufacturer’s protocol. The maximal intra-assay coefficient of variability (CV) 5–6.2% and inter-assay CV 11.3–8.1%. Plasma irisin concentration was also determined before and after exercise by immunoassay method using Elisa Kit (Phoenix Pharmaceuticals Inc., Germany, catalog no. EK06729) according to the manufacturer’s protocol. The maximal intra-assay CV was 4–6% and inter-assay CV was 8–10%.

### Biochemical Assays and HOMA Assessment

Serum glucose was measured by the colorimetric enzymatic method using the diagnostic kit of PZ Cormay S.A. (Warsaw, Poland). The sensitivity of the method was 0.03 mmol⋅L^–1^, the intra-essay CV was 2.20% and the inter-assay CV was 2.10%. The serum insulin concentration was analyzed with an immunoradiometric assay based on coated-tube separation (INS-IRMA kit, Biosource, Belgium). The sensitivity of the method was 1 μIU⋅mL^–1^, the intra-essay CV was 1.9% and the inter-assay CV was 6.3%. Both methods were previously described by Kusy et al. (2013).

The updated computer HOMA2 model was used to determine the β-cell function (HOMA-%B), insulin sensitivity (HOMA-%S), insulin resistance (HOMA-IR) from paired fasting glucose and insulin level. The normal values are 100% for HOMA-%B and HOMA-%S and 1.0 for HOMA-IR (Wallace et al., 2004). The HOMA-indexes were obtained by the software HOMA 2 Calculator, version 2.2.3, copyrighted by The University of Oxford^1^.

Total cholesterol (TCh) was assessed by the reaction of cholesterol esters with cholesterol esterase and oxidase. Low-density lipoprotein cholesterol (LDL-c) was determined with phosphotungstic acid and magnesium ions. High-density lipoprotein cholesterol (HDL-c), remaining in the supernatant, was assessed enzymatically. Triglycerides (TG) assay was based on the reaction with glycerophosphate oxidase. The sensitivities of the methods were 0.026, 0.10, and 0.023 mmol⋅L^–1^ for TCh, HDL-c and TG, respectively. Intra-/inter assay CVs were 1.7/1.7%, 1.20/0.93% and 1.4/1.6%, respectively. The method was also previously described in detail by Kusy et al. (2013).

Lactate (LA) in whole blood (20 μL) was assayed using the spectrophotometric enzymatic method (Biosen C-line, EKF Diagnostics, Barleben, Germany).

### Statistical Analysis

Statistical analyses were performed using the Statistica v.13. software package. The values were presented as mean values ± standard error (SEM). The baseline differences between groups were tested using the Student *t*-test. The differences between values before and after exercise were tested using the two-way repeated-measures ANOVA. If a difference was detected in the ANOVA model, significant differences were determined using Tukey’s *post hoc* test. The results were considered statistically significant when *p* ≤ 0.05. A Pearson product-moment correlation coefficient was computed to assess the relationship between the obtained results.

## Results

### Participant Characteristics

There were no significant between-group differences in demographic and anthropometric characteristics. The exception was the amount of the fat mass. The higher level was observed among untrained participants. The trained group had higher values of physical capacity indicators in the untrained group a good level of aerobic capacity and fatigue tolerance was shown. As expected, significantly higher insulin concentration, HOMA-%B and HOMA-IR but lower HOMA-%S were observed in the untrained group. The concentration of resting glucose did not differ between groups (Table 1).

### Resting Values

At rest, we observed statistically significant differences in the erythrocyte energy status between the trained and untrained groups (Table 2). The ATP level, ATP/ADP ratio and the AEC value were significantly higher in trained than in untrained participants. These results were reflected in the significant positive correlation between erythrocytes energy status and VO_2__*max*_ (*r* = 0.83; *p* = 0.001 for ATP; *r* = 0.61; *p* = 0.003 for ATP/ADP ratio; *r* = 0.65; *p* = 0.001 for AEC) for the combined group of participants. We also noted a significantly higher Hx plasma concentration in the untrained group. Moreover, Hx concentration was negatively correlated with VO_2__*max*_ (*r* = −0.84; *p* = 0.001) and ATP level (*r* = −0.87; *p* = 0.001). The UA concentration did not differ between the groups and was independent of the participants’ VO_2__*max*_. A significantly higher concentration of plasma BDNF was noted in the trained compared to the trained group. The serum irisin concentration tended to be higher in the untrained compared to the trained group, which was supported by a negative correlation between irisin and VO_2__*max*_ (*r* = −0.44; *p* = 0.004). At rest, irisin correlated positively with glucose (*r* = 0.43; *p* = 0.047), insulin (*r* = 0.46, *p* = 0.032) and HOMA-IR (*r* = 0.52; *p* = 0.012) in both groups. Also, erythrocyte ATP level correlated with the parameters of insulin resistance parameters: a negative correlation with insulin (*r* = −0.83; *p* = 0.001) and HOMA-IR (*r* = −0.81; *p* = 0.001) was observed for pre-exercise values.

**TABLE 2:** Red blood cell adenine nucleotides (ATP, ADP, and AMP), cell energy status (AEC, ATP/ADP, and ADP/AMP), plasma purine metabolites (Hx, UA), myokines and lactate in control and trained group at baseline and 10 min after acute exercise.

**Variables**	**Group**	**Baseline**	**After exercise**	***p*-value (Time change)**
ATP RBC (umol⋅L^–1^)	Control	1394.728.4	1429.926.9	0.001
	Trained	1610.330.0*	1662.026.0*	0.001
ADP RBC (umol⋅L^–1^)	Control	213.11.9	195.22.6	0.001
	Trained	179.46.0*	166.25.5*	0.001
AMP RBC (umol⋅L^–1^)	Control	17.60.3	16.30.2	0.001
	Trained	15.90.4*	14.90.4*	0.001
ATP/ADP	Control	6.60.2	7.30.2	0.001
	Trained	9.00.2*	10.10.2*	0.001
ADP/AMP	Control	12.10.1	12.00.1	Ns
	Trained	11.20.1*	11.20.1*	Ns
AEC	Control	0.920.1	0.930.1	0.001
	Trained	0.940.1*	0.950.1*	0.001
Hx (umol⋅L^–1^)	Control	5.10.3	34.62.1	0.001
	Trained	1.40.2*	27.11.4*	0.001
UA (umol⋅L^–1^)	Control	317.88.8	421.39.1	0.001
	Trained	313.56.3	381.48.3*	0.001
Irisin (ng⋅mL^–1^)	Control	9.01.7	7.31.0	Ns
	Trained	6.10.2	6.80.5	Ns
BDNF (ng⋅mL^–1^)	Control	17.20.2	16.80.1	Ns
	Trained	20.80.2*	16.20.5	0.02
LA (mmol⋅L^–1^)	Control	1.00.3	7.51.6	0.001
	Trained	1.10.2	7.91.4	0.001

Values are given as mean ± SE. *p < 0.05, significant difference between trained and control groups at indicated time point. Ns, non-significant differences between indicated time points; ATP, adenosine 5′-triphosphate; ADP, adenosine 5′-diphosphate; AMP, adenosine 5′-monophosphate; AEC, adenylate energy charge; ATP/ADP, adenosine-5′-triphosphate/adenosine-5′-diphosphate ratio; ADP/AMP, adenosine-5′-diphosphate/adenosine-5′-monophosphate ratio; Hx, hypoxanthine; UA, uric acid; BDNF, brain derived neurotrophic factor; LA, lactate. AEC (adenylate energy charge) was evaluated according to the formula by Atkinson AEC = [(ATP) + 0.5(ADP)] / [(ATP) + (ADP) + (AMP)].

### Exerkines Modified by a Single Bout of Intense Exercise

The single bout of exercise did not cause any significant changes in irisin serum concentration in either group (Table 2). Some non-significant tendencies were noted: irisin slightly (18.4%) decreased in the untrained group and increased (12.5%) in the trained group in response to exercise. BDNF blood concentration significantly decreased in the trained group while in the untrained group no detectable changes were observed. Moreover, we observed a significant negative correlation between BDNF and LA in the combined group (*r* = −0.35; *p* = 0.021) but this relationship was more pronounced in the trained group (*r* = −0.56; *p* = 0.004).

### Erythrocyte Purine Nucleotides After Exercise

The single session of an acute bout of exercise induced a significant increase in ATP erythrocyte levels in both trained and untrained groups (Table 2), still, the level was higher in the trained group. Erythrocyte ADP and AMP levels decreased after exercise in both groups. The drop in ADP and AMP levels was accompanied by a significant increase in AEC in both groups. The exercise performed induced an increase in the ATP/ADP ratio in both trained and untrained groups. The ATP level, ATP/ADP ratio and AEC value in erythrocytes after exercise were also positively associated with VO_2__*max*_ among all participants (*r* = 0.83; *p* = 0.001; *r* = 0.54; *p* = 0.01; *r* = 0.59; *p* = 0.004, respectively).

### Post-exercise Changes in Purine Degradation Products

The applied incremental exercise induced a significant increase in Hx concentration in both trained and untrained groups (Table 2). We observed a negative correlation between Hx concentration and VO_2__*max*_ (*r* = −0.79; *p* = 0.001) and erythrocyte ATP levels (*r* = −0.79; *p* = 0.001) after exercise. Moreover, the single bout of exercise elicited an increase in UA concentration in both trained and untrained groups. The latter group had higher UA and Hx concentration after exercise compared to trained participants. The ATP level (*r* = −0.69; *p* = 0.001) and VO_2__*max*_ (*r* = −0.72; *p* = 0.001) after exercise correlated negatively with UA.

### Correlations of Exerkines With Purine Nucleotides

A significant positive correlation was observed between resting BDNF and two indicators of erythrocyte energy status, ATP/ADP ratio (*r* = 0.52; *p* = 0.012) and AEC (*r* = 0.5; *p* = 0.016) in the combined group of participants. Moreover, before exercise, we observed a negative correlation between BDNF and ADP level in erythrocytes (*r* = −0.45; *p* = 0.03). Also, purine degradation product Hx correlated with BDNF at rest (*r* = −0.46; *p* = 0.031). Likewise, irisin correlated with erythrocyte energy status at baseline as confirmed by the significant negative correlation between irisin and AEC (*r* = −0.46; *p* = 0.032). We also observed a positive correlation between irisin and AMP (*r* = 0.48; *p* = 0.025) and Hx (*r* = 0.46; *p* = 0.031). After exercise, we did not observe any correlations between exerkines and purine nucleotides. The correlations are shown in Table 3.

**TABLE 3:** The correlations between exerkines, purine nucleotides and its derivatives and physical performance before exercise.

	**BDNF (ng⋅mL**^–^**^1^)**	**Irisin (ng⋅mL**^–^**^1^)**	**ATP RBC (umol⋅L**^–^**^1^)**
**Red blood cell adenine nucleotides and degradation products**			
ATP RBC (umol⋅L^–1^)	0.37	–0.37	1.00
ADP RBC (umol⋅L^–1^)	−0.45*	0.34	–0.28
AMP RBC (umol⋅L^–1^)	–0.34	0.48*	–0.18
ATP/ADP	0.52*	–0.42	0.70*
AEC	0.51*	−0.46*	0.75*
Hx (umol⋅L^–1^)	−0.46*	0.46*	−0.87*
UA (umol⋅L^–1^)	0.20	0.22	–0.29
**Physical performance**			
VO_2__*max*_ (ml⋅kg^1^⋅min^1^)	0.44*	−0.44*	0.83*
Training hours/week	0.51*	–0.38	0.72*
LA (mmol⋅L^–1^)	−0.35*	–0.08	0.20

Values represent a Pearson product-moment correlation coefficient. *p < 0.05, statistically significant correlation; ATP, adenosine 5′-triphosphate; ADP, adenosine 5′-diphosphate, AMP – adenosine 5′-monophosphate; AEC, adenylate energy charge; ATP/ADP, adenosine-5′-triphosphate/adenosine-5′-diphosphate ratio; Hx, hypoxanthine; UA, uric acid; BDNF, brain-derived neurotrophic factor; VO_2max_, relative maximal oxygen consumption; LA, lactate.

## Discussion

In this study, we have shown for the first time that the training and long-term training adaptation (38 ± 6.0 years of training experience) modifies exerkines blood concentration, purine nucleotides level in erythrocytes and purine degradation products in middle-aged individuals. Resting and post-exercise plasma exerkines (BDNF and irisin) and purine nucleotides levels substantially differed in the highly trained group in comparison to untrained participants. To evaluate if training status has an impact on the above indicators, we assessed their concentration in two conditions: at rest and after acute exercise. Since the effect of chronic exercise training on resting BDNF concentrations is not clear, we decided to involve a group of participants with exceptional training experience (Dinoff et al., 2016). Obtained data revealed higher resting erythrocyte energy status, expressed as ATP level, ATP/ADP ratio and adenylate energy charge (AEC), in the trained than untrained individuals. What is more, a positive correlation between erythrocyte energy status and VO_2__*max.*_was noted. This suggests that higher erythrocyte energy status leads to better oxygen delivery to working muscles in the conditions of increased oxygen demand during maximal physical exercise. Thus, ATP level, ATP/ADP ratio and AEC could be significant indicators of adaptation to a single acute session of exercise.

Purine nucleotide degradation products (Hx and UA) are considered as training status indicators. In this study, significant differences in Hx and UA concentrations between trained and untrained individuals were recorded at rest and after exercise. A negative correlation between these two parameters and VO_2__*max*_ was also observed among all participants after exercise. This result is in line with previously demonstrated data that purine degradation products concentration at rest and after exercise are related to the training status and physical capacity (Saiki et al., 1999; Zielinski et al., 2019). Moreover, both a long-lasting sprint and endurance training lead to a decrease in plasma Hx and UA concentrations (Zielinski and Kusy, 2012, 2015). This suggests that chronic training causes a reduction in muscle adenine nucleotide pool loss and hence, improves muscle adaptation. Such an affect was also observed after a single acute bout of exercise (Zielinski et al., 2019).

Our study aimed to assess whether irisin affects erythrocyte energy metabolism. At baseline, we observed a significant negative correlation between irisin and AEC and a positive correlation between irisin and AMP. These results suggest that there is an association between irisin and erythrocyte energy status as well as metabolism. So far, there have been no studies on an association between irisin and purine degradation products. However, we observed a positive correlation between irisin and Hx, suggesting for the first time a potential association between irisin and regulation of muscle adenine nucleotide pool loss and thus, muscle training adaptation. These phenomena may be related to better training adaptation and higher muscle capacity in trained individuals, reflected by lower irisin concentration, higher AEC value and lower Hx concentration. Animal and human studies showed that irisin has an impact on energy metabolism and ATP level in muscle tissue, prefrontal brain cortex and astrocyte cells. Wang and Pan proved that irisin administration at a concentration of 100 ng/mL improved energy metabolism in the prefrontal cortex by enhancing ATP level. They observed an increase in the activity of mitochondrial complexes I, II, and IV and an up-regulation of ATP level in astrocyte cells via the AMPK pathway (Wang and Pan, 2016). Only in one human study, was an association between irisin and ATP level observed. Huh and coworkers noted that when ATP concentration in muscle decreases, irisin production is upregulated. This relation was observed after a sprint exercise. Authors suggested that irisin could play a role in regulating muscle metabolism and muscle metabolite content (Huh et al., 2012). In our study, we didn’t observe any association between irisin and erythrocyte energy metabolism, thus future studies on larger populations are needed.

Jedrychowski et al. (2015) indicated that healthy humans have circulating irisin levels in the range between 3 and 5 ng⋅mL^–1^, and that these values increase with exercise. In our study, the values measured in the trained group were similar to the recommended range, whereas in the untrained group an elevation of irisin levels was noted. What is more, the study by Huh et al. (2014) revealed that a baseline concentration of irisin was lower in physically active compared to sedentary subjects and noted a negative correlation between baseline values of irisin and VO_2__*max*_. Literature data also indicate that a higher concentration of plasma irisin is observed in pre-diabetes middle-aged males, compared with controls (Norheim et al., 2014). The same tendency was observed in our study, where by a significant negative correlation between irisin concentration and VO_2__*max*_ was obtained. It is noteworthy that after 12 weeks of training, irisin concentration decreased both in pre-diabetic and healthy participants (Norheim et al., 2014). A reduction in irisin concentration related to a response to long-term training, we have observed, is in line with this result. Our study demonstrates that a lower concentration of irisin in trained individuals is associated with higher muscle adaptation, physical capacity and a higher erythrocyte energy status. However, Kerstholt et al. (2015) observed a positive correlation between physical capacity and irisin among female middle-aged subjects, while in males of the same age, the correlation was negative. Therefore, it cannot be excluded that irisin concentration is sex-dependent.

Previous data also showed that irisin is related to glucose metabolism and insulin resistance. Human studies revealed that there is a positive association between circulating irisin concentration and whole-body mass, BMI, fat mass and insulin resistance (Perakakis et al., 2017). The results of our study confirm these data by revealing a significant positive correlation between irisin concentration and fasting glucose, insulin and HOMA-IR. Participants from the trained group, as expected, were characterized by significantly lower insulin concentration and HOMA-IR than the untrained group. Lower insulin resistance was accompanied by lower irisin concentration. An increase in irisin concentration could be related to a compensatory mechanism called the “irisin resistance syndrome,” activated to overcome glucose metabolism disturbances and is associated with insulin resistance (Huh et al., 2015).

Most of the publications so far have focused on changes in irisin concentration in response to physical effort (time, intensity or mode) (Pekkala et al., 2013; Briken et al., 2016; Kabak et al., 2018) but only a few investigated irisin uptake that can modify energetic status. In our participants, irisin was not significantly affected by exercise but different tendencies emerged depending on the training status. A slight decrease in the untrained and an increase in the trained group was noted. Although irisin did not change significantly, we observed a significant post-exercise increase in ATP levels in both groups. The ATP level was higher in the trained group in response to the performed exercise. It was previously found that the erythrocyte ATP level is a regulator of hemoglobin affinity for oxygen. Thus, an increase in ATP level possibly leads to an increase in Hb-A p50 values, resulting in reduced erythrocyte affinity to oxygen and better tissue oxygenation (O’Brien et al., 2015). In the current study, ADP and AMP concentrations post-exercise were significantly lower than before exercise, while ATP/ADP ratio increased. An *in vitro* study indicated that a decrease in ADP level, accompanied by an increase in ATP level and ATP/ADP ratio in erythrocytes, could be caused by a decrease in pH, low pO_2_ with a concomitant rise in intracellular inorganic phosphate-Pi in red blood cells – the conditions characteristic of an intense physical exercise (Berman et al., 1988).

Both regular training and a single bout of exercise are suggested to be the main factors that modify BDNF and irisin concentrations. There have been attempts to elucidate the impact of a single session of exercise on BDNF concentration. A large variety of protocols have been used and subjects of diverse training and health status and age have been involved. Nevertheless, the results remained inconclusive as discordant findings have been revealed (Knaepen et al., 2010). On the other hand, a recently published paper revealed that BDNF concentration increased after regular long-lasting endurance training (Mrowczynski, 2019). We also demonstrated a significantly higher BDNF concentration in the trained group and, what is more, observed a positive correlation between BDNF and VO_2__*max*_. Since, according to the available data, BDNF is also considered as one of the regulators of energy metabolism, we decided to verify if its concentration is related to ATP concentration in erythrocytes. Until now, an association between BDNF and ATP was assessed only in neurons. The study by Cheng et al. (2012) found out that neurons treated with BDNF (20 ng⋅mL^–1^) exhibited an increased ATP production (by about 20%) compared to control neurons, showing that there could be some association between BDNF and energy metabolism. Our study also showed a correlation between BDNF and energy metabolism, this time in human red blood cells. We observed a positive correlation between BDNF and ATP/ADP ratio and adenylate energy charge (AEC). Moreover, a negative correlation between BDNF and Hx was also noted. Both of these associations were observed before exercise. Therefore, BDNF also regulates muscle adenine nucleotide pool loss and muscle adaptation to training. Our results suggest that the higher peripheral BDNF concentration, the higher the erythrocyte energy status and the lower the muscle nucleotide pool loss.

Recently published papers revealed a significant correlation between BDNF and lactate concentration during exercise (El Hayek et al., 2019; Kujach et al., 2019). The results presented by Kemppainen et al. (2005) suggest that with a decreasing glucose supply during a high-intensity exercise, lactate is used by the brain to compensate for the increased energy required to maintain neuronal activity (Kemppainen et al., 2005). In our study, correlation between BDNF and lactate was inverse at rest but absent after exercise. It is worth noting that the experiment by Kemppainen et al. involved young participants, thus we cannot rule out that the training experience of our subjects and the energetic status of their erythrocytes may have compensated for this relationship. Further investigations are still needed. Our work has been partly limited by the factors ranging from the number of subjects involved to the lack of assessment of cognitive function to show an impact of exerkines on cognitive processes. Notwithstanding, the goal of this study was mainly to assess the baseline and post-exercise exerkines concentrations in relation to erythrocyte energy status. Future studies to evaluate cognitive functions and their association with exerkines are essential and needed.

## Conclusion

In conclusion, long-term training results in training adaptation reflected by higher erythrocyte energy status, lower purine degradation products concentration and modified concentration of exerkines (higher BDNF and lower irisin blood concentration). Our study offers an assessment of the energy status in erythrocytes in association with exerkines concentrations in an exceptional cohort of master athletes. It is rare to involve people with such a long training experience in the study, hence, the obtained results are particularly valuable.

## Data Availability Statement

The raw data supporting the conclusions of this article will be made available by the authors, without undue reservation, to any qualified researcher.

## Ethics Statement

The studies involving human participants were reviewed and approved by the Poznań University of Medical Sciences, Decision No. 1252/18. The patients/participants provided their written informed consent to participate in this study.

## Author Contributions

ER and EZ conceived and designed the research and wrote the manuscript. JZ, MK-Z, KK, ER, and EZ conducted the experiments. ER, KK, and MK-Z analyzed the data. EZ, MK-Z, JZ, and KK reviewed and revised the manuscript. All authors read and approved the manuscript.

## Conflict of Interest

The authors declare that the research was conducted in the absence of any commercial or financial relationships that could be construed as a potential conflict of interest.

## Footnotes

1 https://www.dtu.ox.ac.uk/homacalculator

## Abbreviations

ADP: adenosine 5 ′ -diphosphate
AEC: adenylate energy charge
AMP: adenosine 5 ′ -monophosphate
ATP: adenosine 5 ′ -triphosphate
ATP/ADP: adenosine 5 ′ -triphosphate/adenosine 5 ′ -diphosphate
HOMA-IR: homeostatic model assessment
HOMA-%B: β -cell function assessment
HOMA-%B: insulin sensitivity assessment
Hx: hypoxanthine
LA: lactate
RBC: red blood cells
UA: uric acid
VO_2max_: maximal oxygen uptake.
